# Right ventricle and venous system: bridging physiology and clinical practice. A narrative review

**DOI:** 10.62675/2965-2774.20250121

**Published:** 2025-09-09

**Authors:** Suzana Margareth Lobo, Michael R. Pinsky

**Affiliations:** 1 Hospital de Base Faculdade de Medicina de São José do Rio Preto - São José Rio Preto SP Brazil Hospital de Base, Faculdade de Medicina de São José do Rio Preto - São José do Rio Preto (SP), Brazil.; 2 University of Pittsburgh School of Medicine Pittsburgh Pennsylvania United States School of Medicine, University of Pittsburgh - Pittsburgh, Pennsylvania, United States.

**Keywords:** Heart ventricles, Ventricular function, left, Ventricular disfunction, right, Cardiac output, Respiration, artificial, Hemodynamics, Prognosis

## Abstract

The cardiovascular system primarily delivers oxygen and nutrients to tissues. Oxygen delivery depends on cardiac output and arterial oxygen content. While left ventricular function is often emphasized, broader cardiovascular changes, including peripheral vascular function and right ventricular performance, are crucial, especially during shock or cardiopulmonary interactions with mechanical ventilation or fluid challenges. Indeed, the primary role of the left ventricle is to maintain a high central arterial pressure with a minimal filling pressure and to do so efficiently with every beat. Cardiac output is driven by tissue metabolic demand, as feeding arterioles adjust their vasomotor tone to autoregulate blood flow. These adjustments are reflected in proportional changes in venous return to the right ventricle. Right ventricular dysfunction reduces cardiac output primarily by causing systemic venous hypertension, a condition the cardiovascular system is poorly adapted to. Understanding these principles is vital for managing the optimization phase of shock resuscitation. In this narrative review, we aim to provide a comprehensive discussion of the physiological determinants of hemodynamics of circulatory function in shock. This structured yet flexible approach offers an integrative perspective on right ventricular and venous function, highlighting their complexity in hemodynamic regulation.

## INTRODUCTION

The cardiovascular system (CVS) ensures oxygen and nutrient delivery according to tissue metabolic demand, with cardiac output (CO) determined by both left and right heart performance. While clinical focus often centers on left ventricular function, right ventricular (RV) performance and venous return (VR) play equally vital roles, particularly during shock and cardiopulmonary interactions. As conceptualized initially by Guyton and further detailed by Katz, VR and RV function are key determinants of CO and circulatory homeostasis.^(1,2)^ This review aims to provide a comprehensive discussion of the physiological determinants of hemodynamics of circulatory function in shock.

### Ventricular function

Cardiac output is usually quantified as the volume of blood ejected from the heart per unit time (L/minute) and is equal to the product of heart rate (HR) and right or left ventricular stroke volume (SV).

Afterload, the force against which the left ventricle (LV) ejects is closely linked to aortic pressure and wall stress and is primarily influenced by arterioles, which regulate arterial tone and mean arterial pressure (MAP).^(1,2)^ Systemic hypotension impairs effective oxygen delivery to the tissues (DO_2_) by reducing perfusion pressure. Tissue perfusion pressure (TPP) is the difference between MAP and arterial/arteriolar critical closing pressure (Pcc) and ideally should be > 35mmHg.^(3,4)^

Local arterial flow increases with increasing local metabolic demand by selectively reducing vasomotor tone in feeding vessels of those tissues, lowering local Pcc. Thus, hypotension reduces TPP, abolishing autoregulation. While no universal MAP threshold ensures adequate TPP in septic shock, studies recommend an initial MAP ≥ 65mmHg.^(3,4)^ Clinically, maintaining TPP > 35mmHg with MAP > 65mmHg often restores autoregulation, guiding initial shock resuscitation. However, the back pressure to Pcc is tissue capillary pressure, which must be lower than Pcc for decreases in Pcc to increase blood flow.^(4)^ Venous hypertension can impair tissue perfusion even if MAP and TPP are high.

**Figure 1:**
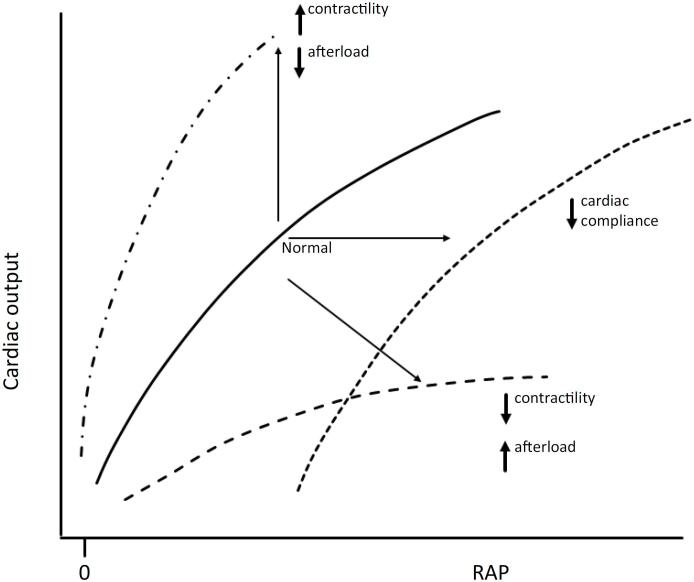
Frank-Starling curves.

Starling's ventricular function curve is the relationship between instantaneous changes in LV end-diastolic volume (LVEDV) and the resultant SV. Alterations in contractility and afterload impact it.^(1,2)^ If one excludes the RV function, one may plot CO *versus* right atrial pressure (RAP) for purposes of illustration (Figure 1). The greater the end-diastolic stretch of the cardiac muscle, the greater the rise in left ventricular SV, in accordance with the Frank-Starling mechanism; simultaneously, RAP decreases, resulting in an upward shift of the VR curve. Both contractility impairment and increased afterload shift the curve downward, reducing CO and raising RAP, while decreased afterload shifts it upward and leftward, enhancing SV and lowering RAP. Diastolic dysfunction or reduced cardiac compliance causes a parallel rightward shift.

RAP - right atrial pressure.

The primary purpose of the Starling mechanism is to instantaneously vary LV contractile function to meet the beat-to-beat changes in LV filling caused by changes in venous flow to the right ventricle (RV) during breathing and at the start of exercise. After increases in LV preload caused by fluid loading or exercise, LVEDV usually returns to its baseline value even if SV remains elevated. This is because homeometric autoregulation (Anrep effect) increases calcium availability by phosphorylation of the calcium channels, transiently increasing contractility.^(5)^ This effect lasts minutes to hours but is unsustainable long-term. This compensatory mechanism may become impaired if critical illness persists beyond a few hours. Chronic ventricular adaptation, characterized by LV dilation and hypertrophy, typically develops over weeks. In acute care settings, transient ventricular dilation may occur as a compensatory response, sometimes associated with improved hemodynamics. In some instances, however, pharmacologic or mechanical support may be temporarily required until stabilization is achieved.

### The right ventricle and the venous system

The primary function of the venous system is to act as a low-resistance; high-capacitance conduit to return blood to the heart and serve as a reservoir for 70 - 75% of the total blood volume (Vt). The venous system has 30 times greater compliance than the arterial system, meaning that blood volume changes usually do not change venous pressure.^(5)^

It is axiomatic that the LV can only eject the amount of blood it receives. Thus, CO must equal the total volume from the venous system, called VR, a function of the RV and venous vascular physiology.^(6,7)^

A two-compartment cardiovascular model illustrates this concept: a central compartment (heart and thoracic vessels) and a systemic compartment that acts as a conduit and blood reservoir.^(8-11)^ The central venous compartment includes the blood volume in the RA, large thoracic, and intra-abdominal veins. Venous return represents blood return from peripheral beds to the thorax and, in steady state, equals CO. Ultimately, when cardiac function is normal, left ventricular CO passively responds to VR rather than driving it. Thus, RV function is critical in maintaining this balance and controlling CO.

Venous return and CO depend on a sufficient decrease in RAP, called central venous pressure (CVP), to generate downstream resistance to VR. Mean systemic filling pressure (MSFP) is the pressure in the venous reservoir, the upstream pressure. Thus, the driving pressure for VR is MSFP-CVP. Under normal conditions, the RV ejects all the blood it receives per beat to keep CVP less than 3mmHg so as not to decrease the driving pressure for VR.^(6)^ Under normal conditions, MSFP is approximately 7 - 10mmHg, creating a VR driving pressure of 3 - 7mmHg. Increased VR temporarily raises CVP, quickly returning to baseline in a beat or two. If CVP rises, VR decreases while CO remains stable until the reduced inflow into the pulmonary circulation reaches the LV about 3 - 4 heartbeats later.^(5,8)^

In summary, RV function adjusts CVP-MSFP to equalize CO and VR.^(5,6)^ Central venous pressure inversely correlates with CO by regulating VR, as small CVP changes during respiration significantly impact VR. The heart can only impair CO by increasing CVP. A normally functioning RV maintains a RAP < 3mmHg such that CO changes are primarily due to changes in MSFP.

### Guyton physiology

According to Guyton, CO is determined by the interaction of two functions: VR, and the heart acting as a pump. This approach begins with some fundamental concepts: MSFP, "stressed" volume (V_S_), "unstressed" volume (V_0_), and resistance to VR (RVR).^(6-12)^

### Mean systemic filling pressure

Cardiac output does not primarily depend on heart function in most conditions, but rather on VR. Ultimately, CVP is the back pressure resisting VR.^(7)^ Thus, if MSFP is the upstream pressure for the venous circulation, while CVP is again the downstream pressure; the driving pressure for VR is MSFP-CVP.^(7,8,10-12)^ Venous return must be inversely proportional to RVR. If systemic arterial pressure was to increase due to rises in α-adrenergic tone, then LV SV would transiently decrease until the immediate increase in SV on the next beat (Starling effect), and if sustained by the Anrep effect, decreasing LVEDV back to its baseline value.^(5)^

Guyton recognized that venous resistance differs from arterial resistance, functioning more as conductance through parallel venous circuits without turbulent flow. Thus, he termed it RVR rather than venous resistance.^(4,13)^ Thus, we can say that VR = (MSFP– CVP)/RVR. The more parallel venous drainage conduits open, the lower the RVR and vice versa.

Understanding MSFP is crucial. Bayliss and Starling theorized that, in the circulation, there must be a point where pressure remains unchanged when the heart stops, reflecting MSFP independent of cardiac function.^(14)^ In simple terms, the heart's pumping maintains the MAP in the aorta at around 80 - 100mmHg. Left ventricle flow out of the aorta and large vessels is slow, sustaining MAP during diastole. Arterial pressure drops in small arteries and arterioles as flow accelerates and vessel diameter decreases. Resistance is proportional to vessel radius to the fourth power, so narrowing arteries with increasing flow causes intravascular pressure to rapidly decrease. Once intraluminal arterial pressure decreases below the vascular wall smooth muscle tone, the vessels spontaneously collapse, stopping flow. When the flow stops, the pressure rises, and the flow resumes only to stop instantly again. This shuttering effect limits blood flow at this Pcc, such that TPP can be defined as MAP - Pcc, allowing tissues to regulate their flow.^(4)^ Tissues regulate their blood flow relative to their metabolic demand by altering arterial vasomotor tone through vascular endothelial feedback. Organ capillary pressures are lower than Pcc, limiting fluid transduction and protecting tissues from high perfusion pressures while maintaining autoregulation. However, sustained increased metabolic demand lowers Pcc, raising capillary pressure, promoting edema, and increasing lymph flow.

For changes in Pcc to affect local blood flow, the MSFP in the tissues must remain below Pcc, establishing the pressure gradient characteristic of a vascular waterfall phenomenon. In both sepsis and vasoplegia, reduced vasomotor tone lowers Pcc. Concurrently, RV failure can elevate CVP, raise MSFP, and reduce VR, which in turn impairs autoregulation. As a result, tissue hypoperfusion may occur despite preserved CO and MAP, due to the narrowing of the gradient between Pcc and MSFP. Similarly, elevated RAP must proportionally increase MSFP if VR is to remain constant. This leads to interstitial edema and organ system dysfunction in capsulated organs (e.g., kidney, liver, lung).^(15)^

If LV output stops, arterial pressure rapidly decreases as blood drains from the large arterial capacitors into the tissues, and CVP increases, both approaching MSFP, taking about 7 - 10 seconds. This pivotal pressure equals MSFP (generally around 7 - 10mmHg). Mean systemic filling pressure is lower than capillary pressure (10 - 12mmHg), but similar to portal pressure and higher than RAP or there would be no VR.^(16-18)^ Venous pressures range from 7 - 12mmHg in venules to 1 - 2mmHg in the superior vena cava and right atrium, varying anatomically across organs.

Bedside studies estimated MSFP using heart-lung interactions in mechanically ventilated patients.^(18-29)^ Inspiratory hold maneuvers at different airway pressures provided transient steady state increased CVP and decreased CO, allowing construction of VR curves. Mean systemic filling pressure was extrapolated as the zero-flow CVP (x-intercept). Other bedside methods, including arm occlusion and mathematical models, have also been validated.^(30-32)^

### Stressed and unstressed volumes

The determinants of MSFP are blood volume and vasomotor tone, while the dynamic determinants are flow distribution and venous conductance (1/R). Approximately 70 - 80% of the blood volume is on the venous side of the pulmonary and systemic circulation, with about 3/4 in small veins and venules and 30% in the splanchnic system.^(6)^ The venous compartment, a distensible blood reservoir, contains Vt, composed of unstressed (V_0_) and stressed volume (V_S_).^(12)^

The V_0_ fills the venous system without altering transmural pressure or MSFP, allowing veins to expand without stretching.^(9-11)^ Once this limit is exceeded, venular stretch increases MSFP as blood shifts from V_0_ to V_S_. Additional volume expands the system, raising elastic pressure on the vessel wall, which is V_S_. Stressed volume influences VR by affecting MSFP, which is directly proportional to V_S_ and inversely proportional to venous compliance. However, when described as flow, all the V_0_ and V_s_ blood behaves as a common reservoir fluid.

Magder et al. found V_S_ averaged 20.2 ± 1.0mL/kg, about 30% of predicted blood volume in patients undergoing hypothermic circulatory arrest.^(28)^ Thus, a large V_0_ reserve can be recruited to sustain MSFP. Blood redistribution or increased vasomotor tone can recruit V_0_ to sustain MSFP. Sympathetic activation or fluid resuscitation shifts V_0_ to V_S_, mobilizing VR during increased metabolic demands like exercise or the ‘fight-or-flight’ response.^(9,10,17)^

### Venous return and resistance to venous return

Vascular resistance to flow is directly proportional to blood viscosity (η) and vessel length, but inversely proportional to the fourth power of vessel radius.^(l)^ The vast cross-sectional area of the vena cava and large vessels contribute little to resistance and primarily serve as volume reservoirs and conductors of flow. There are only a few ways to directly influence VR: affecting MSFP via V_t_, V_S_, and V_0_, or modifying RVR, altering both the position and the slope of the VR curve.^(9-12)^

**Figure 2:**
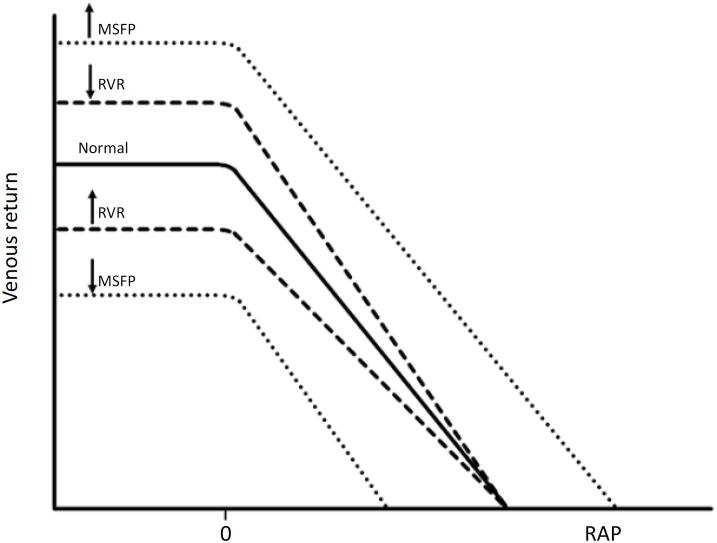
Factors influencing venous return curves.

Therapeutic interventions that modify V_t_ or V_S_ shift the VR curve right or left by altering MSFP, while changes in RVR affect the curve's slope (Figure 2). Resistance to venous return rises when more flow is directed to the gut and abdominal viscera, encountering two resistor circuits: arterial Pcc and hepatic resistance. In contrast, diverting the flow of the muscles during exercise reduces V_0_ and also causes VR to pass through the low-resistance superior and inferior vena cava.

MSFP - mean systemic filling pressure; RVR - resistance to venous return; RAP - right atrial pressure.

### Venous return and cardiac output curves

**Figure 3:**
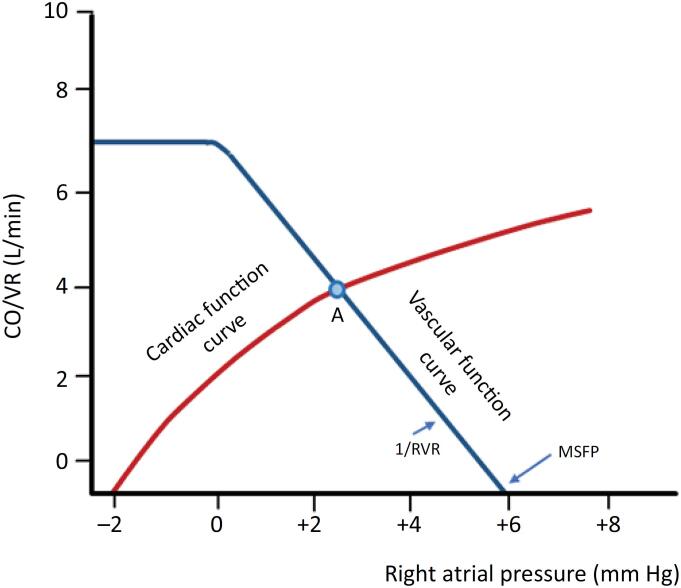
Venous return and cardiac output plotted on the same graph. Relationship between right atrial pressure on the X-axis and VR on the Y-axis. The red curve depicts the cardiac function, and the blue curve represents the vascular function.

Figure 3 illustrates the VR/CO relationship, with RAP/CVP on the X-axis (0 - 7mmHg) and VR on the Y-axis. Venous return increases as RAP decreases but plateaus when RAP falls below atmospheric pressure because large veins collapse at the thoracic entry point when their transmural vascular pressure falls below zero, limiting VR.^(6,7,10)^ This *locus* of vascular collapse is critical for extracorporeal membrane oxygenation (ECMO) catheter placement in an extra-thoracic site, ensuring optimal inflow.

CO - cardiac output; VR - venous return; MSFP - mean systemic filling pressure; RVR - resistance to venous return.

The CVS automatically adjusts to operate at the point where the cardiac function curve and the VR curve intersect (point A). This equilibrium point defines both the CO and VR. A line drawn from point A to the X-axis determines the CVP, usually between 0 - 5mmHg, while a line from point A to the Y-axis shows the VR/CO value.

Venous return reaches its maximum when CVP approaches zero, as this maximizes the pressure gradient between MSFP and CVP. The intersection of the VR curve with the X-axis represents the point where RAP equals MSFP. The slope of the VR curve when RAP > 0 (the diagonal portion) reflects RVR such that the reverse of the RVR slope equals resistance (Figure 3).^(10-12)^

The driving pressure in the arterial system (MAP – Pcc) directly influences the ability of the body to autoregulate blood flow. It indirectly affects CO, while increasing systemic arterial resistance increases MAP, potentially impeding LV ejection. This will increase intrathoracic blood content until a new equilibrium is achieved, and VR and CO become equal again with an elevated LV filling pressure and often an increased RAP. Increased arterial tone shifts the *loci* for Pcc toward the LV and may alter blood distribution by blocking tissue-induced vasodilation. However, only Pcc reductions have been shown to impair peripheral autoregulation. Venous return increases with a larger MSFP-CVP gradient and decreases with higher RVR.

### Physiological changes in shock

Shock states alter MSFP, RVR, and cardiac function through blood loss, venous dilation, or vein obstruction and impaired contractility. They reduce CO and VR by lowering MSFP or VR's driving pressure (MSFP- CVP).

### Hypovolemic shock

**Figure 4:**
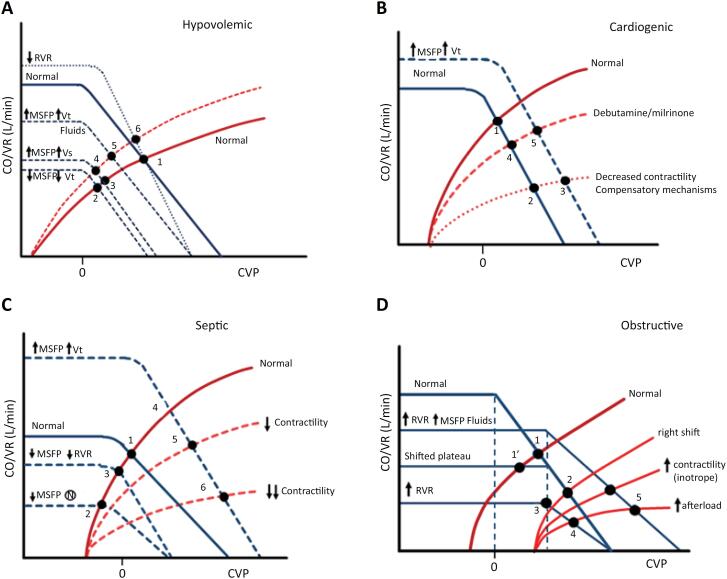
Curves representing the responses of cardiac and vascular function to physiologic and therapeutic interventions in hypovolemic (A), cardiogenic (B), Septic (C), and obstructive shock (D).

The balance between V_S_ and V_0_ can be influenced by physiological responses or therapeutic interventions.^(33-37^) The changes in left-sided (CO) and right-sided (VR) function curves during hypovolemia and hypovolemic shock are shown in figure 4A.^(10,17,18)^ Acute hypovolemia initially decreases V_s_ and induces reflex vasoconstriction through baroreceptor feedback. Hypotension also increases vascular refill from the interstitial space, minimizing the amount of V_0_ needed to restore V_s_ and MSFP. However, with further volume depletion, compensatory mechanisms become insufficient, leading to decompensated hypovolemia, where V_S_ also decreases, VR and CO decline due to decreased MSFP. This results in a leftward shift of the VR curve without a change in slope (RVR remains unchanged), moving cardiac function from point 1 to point 2 (Figure 4A). In addition, sympathetic stimulation mobilizes V_0_ to counteract hypotension via baroreceptor-mediated mechanisms and vasoconstrictors such as alpha-agonists and angiotensin, altering the V_S_/V_0_ ratio (point 2 to 3) without affecting vascular compliance (C), effectively acting as an auto-transfusion. Endogenous catecholamines result in a leftward shift of the ventricular function curve and move point 3 to 4.

CO - cardiac output; VR - venous return; MSFP - mean systemic filling pressure; RVR -resistance to venous return; RAP - right atrial pressure.

The primary strategy to enhance VR in hypovolemic shock is fluid administration to increase Vt and Vs, thereby raising MSFP. This results in an upward shift of the VR curve and a leftward shift of the cardiac function curve (shifts from point 4 to 5, depending on cardiac function). This maintains near-normal CO with moderate blood loss (< 15%). Fluid therapy also decreases blood viscosity, steepens the VR slope with a decrease in RVR, and restores CO/VR. Later, vasodilation from circulating mediators (point 5 to 6) may counteract this effect. More viscous solutions like blood derivatives may induce the opposite effect.

### Cardiogenic shock

Systolic dysfunction flattens the Starling curve, leading to an increase in CVP and a decreased VR gradient. The equilibrium point shifts downward to the right (Figure 4B. point 1 to 2).^(9)^ Observe that at point 2, VR/CO is markedly reduced, and MSFP (the intercept of the VR curve with the x-axis) remains unchanged. Compensatory sympathetic activation slightly increases MSFP to enhance CO with no change in cardiac function (point 2 to 3).^(37)^

The primary effect of dobutamine or milrinone is to improve CO by increasing contractility, reducing RAP, and causing vasodilation with slight MAP and RVR decrease, which increases the VR curve slope, partially offset by a decrease in the V_S_/V_0_ ratio, which reduces MSFP (point 3 to 4).^(11)^ The overall effect is a substantial increase in VR/CO with a concomitant decrease in RAP.

Assuming that V_S_ and MSFP are maintained or augmented with modest fluid support, the intersection of CO/VR curves moves upward toward normal even if contractility remains depressed (point 4 to 5). Excessive volume expansion might not significantly improve VR due to the flat Starling curve. Pure vasoconstrictors tend to increase ventricular afterload, decrease VR/CO, and increase RAP and related filling pressures (shifting the cardiac function curve downward from point 5 to 6). This detrimental effect can be significant if LV function is already impaired or an excessive MAP is targeted.

### Distributive shock

Profound venous vasodilation increases venous capacitance with a subsequent shift of V_S_ to V_0_, decreasing MSFP, while capillary leakage further exacerbates volume loss as seen in septic shock (Figure 4C, point 1 to 2).^(11,18,23)^ However, there is some compensation due to the dilation of large veins and shunting of arterial blood flow to low resistance vascular beds, resulting in a reduction of RVR with the operating point of cardiac function increasing (point 2 to 3). Importantly, Pcc often decreases to values not dissimilar to MSFP, thus eliminating the vascular waterfall essential to allow for autoregulation of blood flow. After fluid resuscitation and vasopressors, a classical hyperdynamic (high CO/Low MAP) hemodynamic state with increased V_S_ and MSFP (point 3 to 4).^(23)^ If there is myocardial depression following fluid challenging or excess use of vasopressors, the CO curve will be displaced to the right (points 4 to 5 and 5 to 6).

### Obstructive shock

Obstructive shock occurs when physical impediments within the CVS hinder VR or impede CO, despite an adequate intravascular volume and intrinsic contractility. Common causes include cardiac tamponade, tension pneumothorax, and pulmonary embolism. The MSFP may remain unchanged or increase due to compensatory vasoconstriction in this condition. However, the obstruction elevates RVR, CVP rises, diminishing the driving pressure for VR and causing a decline in CO.^(11)^ The CVS lacks compensatory mechanisms for acute obstructive shock. Hypotension worsens contractility, and rising CVP further impairs VR. Excessive volume may overdistend the failing RV, worsening LV diastolic compliance, rapidly spiraling to cardiovascular collapse and death within minutes.

Tension pneumothorax (Figure 4D) reduces VR due to increased intrathoracic pressure, which raises pleural pressure (P_PL_) and CVP and impairs cardiac compliance.^(11)^ As P_PL_ increases, the cardiac transmural pressure gradient narrows, compromising diastolic ventricular filling and shifting the ventricular function curve rightward. Although ventricular filling is maintained, it requires a higher CVP, transitioning cardiac function from point 1 to 2. The rise in P_PL_ also compresses the thoracic systemic veins, increasing RVR and further flattening the VR curve, leading to a decline in VR/CO (point 2 to 3). Additionally, lung collapse and hypoxemia increase pulmonary vascular resistance, elevating RV afterload. In response, sympathetic activation raises MSFP via venoconstriction to sustain VR, but this ultimately worsens the decline in VR/CO (point 3 to 4). Treatment strategies should be focused on relieving obstruction, optimizing volume status (point 4 to 5), and supporting cardiac function with inotropes (point 5 to 6). However, VR can only be restored by resolving the mechanical obstruction.

### Fluid and vasopressors interactions in shock

**Figure 5:**
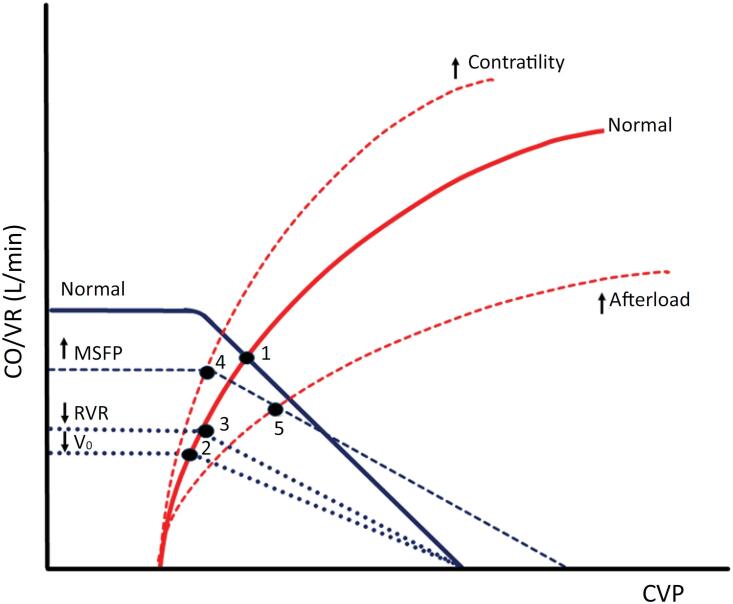
Response to vasopressor in fluid-responsive patients during distributive shock.

An individualized, physiology-based approach to fluid and vasopressor management, guided by the patient's hemodynamic profile, is warranted.^(34,35,38)^ Visualizing CO/VR curves helps us to understand how fluids and vasopressors interact during the resuscitation of distributive shock (Figure 5). During vasodilation, for instance, after anesthesia induction with propofol, the sudden increase in venous capacitance converts V_S_ into V_0_, decreasing MSFP and VR/CO.^(39)^ The cardiovascular function operating point shifts from point 1 to point 2. Simultaneously, dilation of large veins and the diversion of arterial blood flow to low-resistance vascular beds also reduce RVR, steepening the VR curve and shifting the operating point from 2 to 3.

CO - cardiac output; VR - venous return; MSFP - mean systemic filling pressure; RVR - resistance to venous return; V0 - non-stressed volume.

Vasopressors constrict both arteries and veins, increasing RVR without altering MSFP. However, by constricting small veins, they mobilize unstressed blood volume, acting like a fluid bolus functionally equivalent to up to one liter of crystalloid.^(28)^ This reduction in venous capacitance increases the proportion of V_S_ to V_0_, raising MSFP and partially offsetting the VR decline. Consequently, the VR curve shifts rightward, meaning that in fluid-responsive patients, vasopressors enhance MSFP and thus VR/CO. However, high doses of vasoconstrictors increase ventricular afterload, shifting the curve downward (point 4 to 5), reducing VR/CO.

Norepinephrine increases CO by enhancing cardiac preload. Patients with early life-threatening sepsis-induced hypotension requiring norepinephrine underwent hemodynamic measurements. Cardiac output significantly increased, accompanied by a rise in global end-diastolic volume, a preload surrogate, and a decrease in SV variation from 13 to 9%.^(29)^ These findings suggest that CO increases after NE administration result from both enhanced preload (a "fluid-like" vasopressor effect) and improved contractility. In preload-responsive patients, NE effectively raises cardiac preload, boosting CO. They also noted that early NE administration enhanced diastolic arterial pressure, improving left ventricular coronary perfusion. In patients with high MAP and low ejection fraction (< 45%), NE had neutral but not deleterious hemodynamic effects.

The role of potentiating fluid expansion by reducing V_0_ and increasing Vs, thereby raising MSFP, was studied in septic shock patients.^(20)^ The researchers tested whether MSFP (estimated by CI/CVP curves) changes induced by volume challenges were greater at higher norepinephrine doses. Norepinephrine doses were adjusted to target MAP, and MSFP changes were measured during passive leg raising at high and low norepinephrine doses. Passive leg raising-induced increase in MSFP (delta MSFP) is larger at a higher dose of NE compared to a lower dose. Therefore, early NE initiation may prevent fluid overload and expedite shock reversal by achieving target MAP and CO with less fluid.

Several studies suggest that earlier NE administration may improve outcomes by reducing the volume required for resuscitation.^(20,38-44)^ The efficacy of fluid resuscitation is enhanced by NE, producing effects that are both additive and potentiated. This occurs because fluid volume is distributed within a more constricted venous network, amplifying its impact. Optimizing MSFP with fluids and vasopressors will improve VR and tissue perfusion. Positive pressure ventilation by inducing hyperinflation may increase RV afterload, potentially leading to dysfunction, especially in patients with compromised cardiovascular reserves.^(45-48)^ Furthermore, the therapy should be adaptive to the evolving physiology of each patient during the different phases of shock.^(49)^

## Final remarks

The physiology of venous return is remarkably intricate, characterized by multiple independent variables that interact asynchronously. Understanding these dynamics is essential for effective shock management. The complexities of both physiological and pathophysiological responses defy simplification; the accompanying graphs are intended solely for educational purposes.
